# Incidence of healthcare-associated infections with invasive devices and surgical procedures in Nepal

**DOI:** 10.5588/pha.21.0039

**Published:** 2021-11-01

**Authors:** P. Koju, X. Liu, R. Zachariah, M. Bhattachan, B. Maharjan, S. Madhup, H. D. Shewade, A. Abrahamyan, P. Shah, S. Shrestha, H. Li, R. Shrestha

**Affiliations:** 1 Dhulikhel Hospital, Kathmandu University Hospital, Dhulikhel, Nepal; 2 School of Health Sciences, Global Health Institute, Wuhan University, Wuhan, China; 3 School of Health Sciences, Faculty of Biology, Medicine and Health, University of Manchester, Manchester, UK; 4 UNICEF/UNDP/World Bank/WHO Special Programme for Research and Training in Tropical Diseases (TDR), World Health Organization, Geneva, Switzerland; 5 World Health Organization, Country Office, Kathmandu, Nepal; 6 International Union Against Tuberculosis and Lung Disease (The Union), Paris, France; 7 The Union South East Asia, New Delhi, India; 8 Tuberculosis Research and Prevention Centre, Yerevan, Armenia

**Keywords:** SORT IT, operational research, surveillance, health systems, Sustainable Development Goals, infection prevention and control

## Abstract

**SETTING:**

Dhulikhel Hospital, Kathmandu University Hospital, Kathmandu, Nepal.

**OBJECTIVES:**

1) To report the incidence of health-care-associated infections (HAIs), 2) to compare demographic, clinical characteristics and hospital outcomes in those with and without HAIs; and 3) to verify bacterial types in HAI and community-acquired infections (CAIs) among inpatients with invasive devices and/or surgical procedures.

**DESIGN:**

This was a cohort study using secondary data (December 2017 to April 2018).

**RESULTS:**

Of 1,310 inpatients, 908 (69.3%) had surgical procedures, 125 (9.5%) had invasive devices and 277 (21.1%) both. Sixty-six developed HAIs (incidence = 5/100 patient admissions, 95% CI 3.9–6.3). Individuals with HAIs had a 5.5-fold higher risk of longer hospital stays (⩾7 days) and a 6.9-fold risk of being in intensive care compared to the surgical ward. Unfavourable hospital exit outcomes were higher in those with HAIs (4.5%) than in those without (0.9%, *P* = 0.02). The most common HAI bacteria (*n* = 70) were *Escherichia coli* (44.3%), *Enterococcus* spp. (22.9%) and *Klebsiella* spp. (11.4%). Of 98 CAIs with 41 isolates, *E. coli* (36.6%), *Staphylococcus aureus* (22.0%) and methicillin-resistant *S. aureus* (14.6%) were common.

**CONCLUSION:**

We found relatively low incidence of HAIs, which reflects good infection prevention and control standards. This study serves as a baseline for future monitoring and action.

## Abstract (FR)

**LIEU:**

Hôpital de Dhulikhel, Hôpital Universitaire de Katmandu, Katmandu, Népal.

**OBJECTIFS:**

1) Rapporter l’incidence des infections associées aux soins (HAI), 2) comparer les caractéristiques démographiques et cliniques et les résultats hospitaliers chez les patients atteints et non atteints de HAI ; et 3) vérifier les types bactériens des HAI et des infections communautaires (CAI) parmi les patients hospitalisés avec dispositifs invasifs et/ou ayant subi une intervention chirurgicale.

**MÉTHODE:**

Étude de cohorte réalisée en utilisant des données secondaires (décembre 2017 à avril 2018).

**RÉSULTATS:**

Sur 1 310 patients hospitalisés, 908 (69,3%) ont subi une intervention chirurgicale, 125 (9,5%) avaient des dispositifs invasifs et 277 (21,1%) avaient à la fois un dispositif invasif et subi une intervention chirurgicale. Au total, 66 ont contracté une HAI (incidence = 5/100 admissions de patients, IC 95% 3,9-6,3). Les patients atteints de HAI avaient un risque 5,5 fois plus élevé de séjour prolongé à l’hôpital (⩾7 jours) et un risque 6,9 fois plus élevé d’être admis en soins intensifs qu’en service de chirurgie. Les résultats défavorables à une sortie d’hôpital étaient plus fréquents chez ceux atteints de HAI (4,5%) que chez ceux non atteints de HAI (0,9%, *P* = 0,02). Les bactéries les plus fréquemment responsables de HAI (*n* = 70) étaient *Escherichia coli* (44,3%), *Enterococcus* spp. (22,9%) et *Klebsiella* spp. (11,4%). Sur 98 CAI avec 41 isolats, *E. coli* (36,6%), *Staphylococcus aureus* (22,0%) et *S. aureus* résistant à la méticilline (14,6%) étaient les plus fréquents.

**CONCLUSION:**

Nous avons observé une incidence relativement faible de HAI, ce qui reflète de bons standards de contrôle et de prévention des infections. Cette étude sert de référence à de futures actions et stratégies de suivi.

Healthcare-associated infections (HAIs), otherwise known as nosocomial or hospital-acquired infections, occur during the process of receiving care in a health facility. Such infections include occupational infections among health staff.^1^ HAIs are a recognised global public health challenge affecting over 1.4 million patients, a large proportion caused by antibiotic-resistant organisms.^2^,^3^ The burden of HAIs in lowand middle-income countries (LMICs) has been assessed in systematic reviews.^4^,^5^ One of the reviews reported a pooled prevalence of 15.5 per 100 patients, significantly higher than in high-income countries, where this is situated around five per 100 patients.^4^ The other review, focused on South East Asia and revealed an overall HAI prevalence of 9%.^5^ Use of central lines, ventilators and other invasive devices increase the risk of HAIs by 19 times in LMICs when compared with high-income countries.^6^ Similarly, surgical site infection (SSI) is nine times higher in LMICs and affects about two thirds of operated patients.^6^ HAIs have major public health implications, including prolonged hospital stays, long-term disability, additional costs to health systems, patients and families, increased antimicrobial resistance (AMR) and unnecessary deaths of patients and health workers.^7^

A previous study has shown an HAI incidence of 27.3/1,000 patient days with device-associated infections in an intensive care unit (ICU) of the one of the teaching hospitals in Kathmandu, Nepal.^8^ Another study from the same ICU reported 96% drug resistance among Gram-negative bacterial isolates.^9^ While these studies were focused on ICUs, there has been no study on HAIs from Nepal or neighbouring countries which include ‘all units of a tertiary-level facility’ and its link to hospital exit outcomes.

Such information is vital to reinforce and advocate for infection prevention and control (IPC) measures, as HAIs serve as a proxy indicator of the standard of IPC. Furthermore, knowledge on types of bacteria causing HAIs and how these compare with community-acquired infections (CAIs) would be useful to assess possible spread of AMR between health facilities and the community and visa-versa. These subject areas have also been identified as national research priorities and align well with the strategic pillars of the global and national action plans on tackling AMR.^6^,^10^

Dhulikhel Hospital, Kathmandu University Hospital, Kathmandu, Nepal, is a major tertiary-level hospital with five intensive care units (ICUs) covering adults, children and neonates. It is also a national AMR surveillance site since 2008. Between 2017 and 2018, comprehensive data on HAIs and their economic burden was collected by a dedicated research team.

Among inpatients who had invasive devices and/or surgical procedures at the Dhulikhel Hospital, we aimed to characterise those with and without HAIs and their hospital exit outcomes. Our specific objectives were 1) to report on the incidence of HAIs/100 inpatient admissions; 2) to compare demographic, clinical characteristics, as well as hospital exit outcomes between those with and those without HAI; and 3) to verify if bacterial profiles in various specimens among those with HAI and CAIs were different.

## METHODS

### Study design

This was a cohort study involving secondary data from a research study evaluating the economic impact of HAIs.^11^

### Setting

Nepal is a low-income country located in South-East Asia and lies in the Himalayas. It is bordered by China and India and has an estimated population of 30.2 million.^12^ In 2015, Nepal became a secular federal parliamentary republic divided into seven provinces.

Dhulikhel Hospital, Kathmandu University Hospital is situated in Dhulikhel Town, Kavre District, 30 km northeast of Kathmandu. The hospital is an independent, not for profit, non-governmental institution. It has 425 beds and covers a population of approximately 1.9 million people from six neighbouring districts.^13^

The hospital has various departments, including outpatient and inpatient services. The latter includes medicine, obstetrics and gynaecology, general surgery, orthopaedics, trauma, ear, nose and throat, dental and intensive care units. It is also a designated regional centre for emergency management of road traffic accidents.

The hospital started its IPC programme in 2010. All hospital staffs are trained in IPC measures and there is an IPC Committee with a dedicated nurse for monitoring IPC activities in accordance with the WHO 2004 guidelines.^14^ Hand hygiene, HAIs and environmental monitoring are all part of hospital IPC surveillance activities. The hospital has an IPC manual for all staffs.^15^

### HAIs related to invasive devices and surgical procedures

HAIs were defined as infections acquired after 48 h of hospital admission and that were not present or incubating at the time of admission.^16^ SSI was defined as infection that occurs within 30 days after surgery or after 90 days of surgical intervention while in hospital or within 1 year after an implant insertion.^17^,^18^ The criteria for the classification of HAIs were adapted from the US Centers for Disease Control and Prevention as shown in Table 1.^19^ CAI was defined as infection developed outside of a hospital and present at the time of admission.^20^ Invasive devices included central lines, intubation tubes, intravenous catheters, urinary catheters and drains.

**TABLE 1:** Criteria and case definition of HAI^19^ and CAI^20^ used in Kathmandu University Hospital, Kathmandu, Nepal, December 2017–April 2018

Type of infection	Definition
CAI	Defined as an infection contracted outside of the healthcare facility or an infection present at the time of admission
HAI	Defined as new infections a) acquired after 48 h of hospital admission were not present or incubating at the time of admission, b) or based on criteria as below
Central line-associated bloodstream infection	Patient has central line in place Patient of any age has at least one of the following signs or symptoms: a) fever (>38.0°C), b) chills, or c) hypotension Organism(s) identified from blood is not related to an infection at another site AND the same is identified from two or more blood specimens drawn on separate occasions
Ventilator-associated infection	Patient must have following: Patient has been in ventilator for ⩾48 h on the date of event Patient must have at least one of following signs and symptoms: a) fever or hypothermia, b) change in secretions, c) cough, d) apnoea/bradycardia, e) tachypnoea Positive cultures of sputum/tracheal aspirate/pleural fluids New or changing infiltrates in X-ray
Catheter-associated urinary tract infection	Patient must have following: Patient had an indwelling urinary catheter that had been in place for >2 days on the date of event AND was either: present for any portion of the calendar day on the date of event, OR removed the day before the date of event Patient has at least one of the following signs or symptoms: a) fever ( >38.0°C), b) suprapubic tenderness (with no other recognised cause), c) costovertebral angle pain or tenderness (with no other recognised cause), d) urinary urgency (when no catheter), e) urinary frequency (when no catheter), f) dysuria (when no catheter) Patient has a urine culture with no more than two species of organisms identified, at least one of which is a bacterium of ⩾10^5 cfu/ml
SSI	Must meet the following criteria: Date of event for infection occurs within 30 days or 90 days after any operative procedure or up to 1 year after implant insertion Patient has at least one of the following: a) purulent drainage from the superficial incision/deep incision/a drain that is placed into the organ/space; b) organisms identified from an aseptically obtained specimen from the wound/incision/fluid or tissue in organ; c) incision that is deliberately opened and organism identified in culture; AND patient has at least one of the following signs or symptoms: fever ( >38°C), localised pain or tenderness; localised swelling; erythema; or heat; d) diagnosis of a SSI

HAI = healthcare-associated infection; CAI = community-acquired infection; cfu = colony forming unit; SSI = surgical site infection.

The HAI monitoring team included three nurses, two microbiologists, one public health professional and a project manager. All staff were trained and a project manager and public health officer supervised the team.

### Sample collection and laboratory analysis

Samples were collected by aseptic technique and sent to the hospital laboratory where culture was performed on nutrient agar, blood agar, MacConkey agar, chocolate agar and cysteine lactose electrolyte deficient agar plates; all the blood samples were first inoculated in BACTEC™ bottles (BD, Franklin Lakes, NJ, USA), followed by sub-culture on solid media. Colonies were sub-cultured for purity and identified by colony morphology and biochemical tests according to standard procedures.^21^

### Study population and period

The study population included all patients ( ⩾1 year of age) admitted to Dhulikhel Hospital between 16 December 2017 to 16 April 2018, and had an invasive device or had undergone surgical procedures during their hospital stay.

### Data collection, sources of data and validation

Data were collected by a research team and included the patient identification number, date of admission, date of discharge, age, sex, hospital ward, type of invasive device, surgical procedures and type of infection (HAI or CAI). Standardised hospital exit outcomes were extracted from the patient files, and included favourable (discharged) and unfavourable outcomes (not improved, discharged against medical advice, referred out for complications, and died).

Data were entered into EpiData software v3.1 (EpiData Association, Odense, Denmark). Data entry, data cleaning and data coding were supervised by the research staff and cross-validated by different individuals through verification of individual patient records with the database.

### Data analysis and statistics

Data were analysed using EpiData analysis v2.2.2.186 (EpiData Association). Differences between those with and those without HAI were compared using the χ^2^ test, and measures of risk were estimated using crude and adjusted relative risks (RRs). RRs were adjusted through log binomial regression using Stata software v12.1 (Stata Corp, College Station, TX, USA). The level of significance was set at *P* ⩽ 0.05 and 95% confidence intervals were used throughout.

### Ethics approval

Permission to conduct the study was received from hospital director. The Institutional Review Committee of Kathmandu University School of Medical Sciences (IRC-KUSMS), Dhulikhel Hospital, Kathmandu, Nepal, approved the initial research study conducted in 2017–201811 (approval number 130/17). The current nested study was based on the same data set with ethics cover. Ethics approval was also obtained from the Ethics Advisory Group of the International Union Against Tuberculosis and Lung Disease, Paris, France (Approval No 03/20, dated 12/2/2020).

## RESULTS

### Characteristics of the study population and incidence of HAIs

A total of 1,310 inpatients were included, of whom 125 (9.5%) had invasive devices, 908 (69.3%) had surgical procedures and 277 (21.1%) had both interventions. Most patients (88.6%) were admitted to the surgical ward, followed by the medical ward (9.9%) and intensive care unit (1.5%). A total of 98 CAIs were diagnosed. There were 66 HAIs, giving an HAI incidence of 5/100 patient admissions (95% CI 3.9–6.3).

### Demographic and clinical characteristics associated with HAIs

Demographic and clinical characteristics of patients with HAIs and associated risk factors are shown in Table 2. Individuals with HAIs had a 5.5-fold higher adjusted risk of longer hospital stays ( ⩾7 days) than those without HAIs. Patients in the intensive care unit had 6.9-fold higher risk of HAIs than those in the surgical ward. For inpatients with HAI, the median hospital stay was 11 days (range 7–18), while for those without HAIs, this was 5 days (range 4–7); this was statistically significant (*P* < 0.001).

**TABLE 2:** Demographic and clinical characteristics associated with HAI among patients who had invasive devices and/or surgical procedures, Kathmandu University, Kathmandu, Nepal, December 2017–April 2018

Characteristics	Total *N*	Patients with HAI		RR	(95% CI)	aRR^*^	(95% CI)	*P* value

		*n*	(%)					
Total	1310	66	(5.0)					
Age, years								
<35	730	32	(4.4)	0.8	(0.5–1.2)	^†^		
⩾35	580	34	(5.9)	1	—			
Sex								
Male	566	31	(5.5)	1.2	(0.7–1.9)	^†^		
Female	744	35	(4.7)	1	—			
Hospital stay, days								
<7	979	20	(2.0)	1	—	—		
⩾7	331	46	(13.9)	6.8	(4.1–11.3)	5.5	(3.2–9.4)	<0.001
Type of ward								
Surgical ward	1160	51	(4.4)	1	—			
Medical ward	130	4	(3.1)	0.7	(0.3–1.9)	0.7	(0.2–1.9)	0.5
Intensive care unit	20	11	(55.0)	12.5	(7.8–20.2)	6.9	(4.6–10.2)	<0.001
Type of intervention								
Invasive device only	125	8	(6.4)	1.9	(0.9–4.1)	0.8	(0.3–1.8)	0.6
Surgical procedure only	908	30	(3.3)	1	—	—		
Both	277	28	(10.1)	3.1	(1.9–5.0)	1.6	(0.9–2.7)	0.1

^*^Log binomial regression.

^†^ Age and sex not included in the regression model as their crude *P* value (association with HAI) was >0.2.

HAI = healthcare-associated infection; RR = risk ratio; CI = confidence interval; aRR = adjusted RR.

### Hospital exit outcomes in relation to HAIs

Among 1,310 patients, hospital exit outcomes were unknown for 25 (2.1%) patients without HAIs. The remaining 1,285 hospital exit outcomes are shown in Table 3. Unfavourable outcomes were significantly higher in those with HAIs (4.5%) than in those without HAIs (0.9%, *P* = 0.02).

**TABLE 3:** Hospital exit outcomes among patients who had invasive devices and/or surgical procedures in relation to HAI, Kathmandu University, Kathmandu, Nepal, December 2017–April 2018

	With HAI		Without HAI		*P* value^*^
	
	*n*	(%)	*n*	(%)	
Total	66		1244		
Unknown	0		25		
Exit outcomes	66		1219		
Favourable (discharged)	63	(95.5)	1208	(99.1)	0.02
Unfavourable	3	(4.5)	11	(0.9)	0.02
Died	1		1		
Left against medical advice	0		1		
Not improved	1		7		
Referred out	1		2		

^*^Fisher’s exact test.

HAI = healthcare-associated infection.

### Bacterial types among those with HAIs and CAIs

Bacterial isolates by specimen type among those with HAIs and CAIs are shown in Table 4. Among 70 isolates cultured from inpatients with HAIs, with the three most common bacterial types were *Escherichia coli* (44.3%), *Enterococcus* spp. (22.9%), and *Klebsi-ella* spp. (11.4%). In those with CAIs, there were a total of 41 isolates cultured of which *E. coli* (36.6%), *Staphylococcus aureus* (21.9%), and methicillin-resistant *S. aureus* (MRSA) (14.6%) were the most common bacteria.

**TABLE 4:** Bacterial isolates among patients who had HAI and CAI, Kathmandu University, Kathmandu, Nepal, December 2017–April 2018

Bacterial isolates by specimen type	HAI *n*	CAI *n*	*P* value
Total	70^*^	41^†^	
Pus	26	18	
*Escherichia coli*	11	3	0.04
*Enterobacter* spp.	0	1	
*Enterococcus* spp.	5	2	
*Klebsiella* spp.	3	0	
MRSA	3	3	
*Pseudomonas aeruginosa*	1	0	
*Staphylococcus aureus*	2	6	0.02
*Streptococcus* spp.	1	3	
Urine	16	12	
*Escherichia coli*	9	10	
*Enterococcus* spp.	4	1	
*Klebsiella* spp.	3	1	
Wound swab	12	5	
*Escherichia coli*	2	0	
*Enterobacter* spp.	1	0	
*Enterococcus* spp.	3	0	
*Klebsiella* spp	2	1	
MRSA	1	2	
*Pseudomonas aeruginosa*	1	0	
*Staphylococcus aureus*	2	2	
Bile	9	—	
*Escherichia coli*	5	—	
*Enterococcus* spp.	3	—	
*Pseudomonas aeruginosa*	1	—	
Sputum	2	3	
*Escherichia coli*	2	0	
*Klebsiella* spp.	0	1	
MRSA	0	1	
*Pseudomonas aeruginosa*	0	1	
Blood *Escherichia coli*	—	2	
Intra-abdominal collection	2	—	
*Escherichia coli*	1	—	
*Enterococcus* spp.	1	—	
Tracheal aspirate	2	—	
*Escherichia coli*	1	—	
*Staphylococcus aureus*	1	—	
CSF *Staphylococcus aureus*	—	1	
Tissue *Streptococcus* spp.	1	—	

^*^ Total bacterial isolates from 66 inpatients.

^†^ Total bacterial isolates from 98 inpatients.

HAI = healthcare-associated infection; CAI = community-acquired infection; MRSA *=* methicillin-resistant *Staphylococcus aureus*; CSF = cerebrospinal fluid.

The number of *E. coli* isolates in pus was significantly higher among patients with HAIs (*n* = 11) than among those with CAIs (*n* = 3). All 11 isolates in HAI were from SSIs, of which nine were linked to abdominal surgeries. The three isolates in CAIs were from abscesses (perineal, appendicular, soft tissue/fasiculitis). The number of *S. aureus* isolates in pus was significantly higher in CAIs (*n* = 6, all from abscesses) than in HAIs (*n* = 2, from SSIs).

## DISCUSSION

This study which assessed HAIs in the medical, surgical and intensive care units of a major tertiary hospital in Nepal shows a relatively low HAI incidence of 5/100 inpatient admissions. Individuals with HAIs had significantly longer hospital stays had less favourable hospital outcomes and those in the ICU were at higher risk of contracting such infections.

The HAI incidence in our study compares well with that reported from high-income countries and is about three-fold lower than in similar LMIC settings.^4^,^5^ Our study findings are important, as the lower HAI incidence serves as a proxy for high IPC standards and quality of care. Since 2010, the hospital management has invested in IPC and this is the likely fruit of such efforts. Another study conducted in the same hospital in 2014 among patients underwent surgery found the SSI to be 2.6%.^22^ The internal assessment conducted in hospital from July 2017 to June 2018 showed respectively 2.7% and 6.0% SSI in the general and gynaecology operation theatres. An internal survey carried out in the surgery department from January to October 2019 found an SSI rate of 2.6% and a rate of 2.4% from January to August 2018 . These were evaluation of inpatients during the time of hospital stay only. At a time when Nepal, like many other countries is struggling with the COVID-19 pandemic,^23^ this is reassuring news, and the hospital teams, particularly those involved with IPC, deserve to be commended. The current study also serves as a useful yardstick for future assessments.

Seasonal variations have been observed with infections.^24^ Few studies found significantly higher incidence of Gram-negative infections and SSIs during summer time.^25^–27 The current study was conducted during winter, which may have influenced the incidence of HAIs; however, we have no data to substantiate this.

The study findings have some policy and practice implications. First, the overall outcomes in both groups (HAI and no HAI) were excellent and exceeded 90%. From a public health perspective, this is commendable and likely to mirror the quality of care and high standards of IPC in the hospital, which needs to be maintained.

Second, 94% of all HAIs were from the surgical and ICU units, with those in the ICU having an almost seven-fold higher risk of contracting HAIs than those in surgical wards. This is understandable, as ICU patients are more exposed to invasive devices and longer hospital stays, thereby increasing their risk of contracting HAIs.^28^,^29^ The ICU and surgical wards are areas where focused attention might yield further dividends in reducing HAI incidence.

Third, *E. coli* was the most common bacteria isolated in those with HAIs and this was significantly higher than in CAIs, which are in concordance with the findings of the study conducted earlier in the same hospital.^22^ Such bacteria were found in pus from abdominal surgery, indicating stool contamination during the intervention. Vigilance in operative procedures and elaborate toileting during abdominal surgery are measures to be considered to reduce residues of accidental faecal contamination. In addition, existing evidence suggests that wound protectors, antibacterial sutures and negative pressure wound therapy are effective in preventing such infections and reducing post-operative complications.^30^ Also, standard pre-operative antibiotic prophylaxis timing is considered useful to reduce post-operative infection rates.^31^ These measures could be considered to reduce contamination and infection.^32^

As regards CAIs, MRSA was observed in 14% of inpatients; this highlights the risk of introducing potentially troublesome and antibiotic-resistant bacteria to the hospital from the community. This potential risk of transmission would be akin to ‘antibiotic resistance in communities is antibiotic resistance in hospitals, and will be antibiotic resistance everywhere’.

Finally, the Kathmandu University Hospital is a WHO sentinel site for AMR surveillance since 2008. This provides an excellent opportunity to continue operational research to monitor HAIs and also conduct new research focused on a better understanding of CAIs. In particular, the patterns of AMR would be relevant to inform antibiotic regimens, including newer-generation antibiotics for HAIs and CAIs.

The study strengths were as follows: we included all the main hospital wards; there were standardised case definitions for diagnosing HAIs and CAIs; all staff were well trained and operated under rigorous research conditions; data were cross-validated with patient records and we adhered to STROBE (Strengthening the reporting of observational studies in epidemiology) guidelines for the conduct and reporting of observational studies in epidemiology.^33^ Furthermore, the study subject addresses an identified national operational research priority and is thus highly relevant.

The main study limitation is that we used an existing database that was designed for an economic evaluation of the impact of HAIs and as such, individuals with little or no financial information have been excluded.^34^ Infants were also excluded. Such exclusions might have negated unfavourable hospital exit outcomes in the HAI group through a ‘healthy cohort effect’. Furthermore, for SSI follow-up time was not available for the 30-day period prior to hospital presentation, 90-day period after a surgical intervention and for the year after implant insertion. As such, we were unable to present incidence rate (hazard ratios) using person-time.

In conclusion, the study has shown a relatively low incidence of HAIs in a tertiary hospital in Nepal, which is reassuring in terms of the acquisition and transmission of AMR. Being ‘a proxy’ for IPC standards, this is welcome news during the COVID-19 pandemic, as it indicates that good IPC standards are in operation. Areas that could merit focused attention include the surgical unit and the ICU. This study also serves as a baseline for future monitoring of HAIs and informing action.
